# Staying Connected to Our Purpose in Nursing: Linking Past Motivations With Current Practice

**DOI:** 10.1111/jan.16974

**Published:** 2025-04-10

**Authors:** Carmel Bond

**Affiliations:** ^1^ Mental Health Nursing Sheffield Hallam University Sheffield UK

## 1 Motivation and Purpose in Nursing

Many individuals enter the nursing profession driven by a deep sense of care and responsibility toward others. Often, personal experiences or observations of healthcare challenges inspire a commitment to providing compassionate person‐centred care. These motivations shape professional journeys, reinforcing the resilience and empathy needed to navigate the complexities of nursing. The role of nurses extends beyond clinical tasks, encompassing emotional support, advocacy, and the ability to respond to diverse patient needs with compassion and competence.

Across generations, those in the nursing profession have found meaning in their work by transforming difficult experiences into opportunities for growth. While each nurse's journey is unique, the common thread of dedication to patient care remains a defining characteristic of the profession. This shared sense of purpose unites nurses across different specialties, cultures, and healthcare systems, creating a global network of committed professionals.

## 2 Adapting and Thriving: Lessons of the Past and the Challenges of the Present

Throughout history, nurses have demonstrated remarkable resilience in the face of adversity. Figures such as Linda Richards (1841–1930), America's first professionally trained nurse, exemplified perseverance in overcoming personal loss to build a distinguished career in nursing. Similarly, Mary Seacole (1805–1881) overcame racial discrimination to provide care during the Crimean War, ensuring that those in need received vital support. Their stories serve as powerful reminders of the resilience required to sustain a lifelong commitment to nursing.

Resilience, often understood as the ability to positively adapt to adversity (Jackson et al. 2007), remains essential in contemporary nursing. Healthcare professionals must navigate demanding work environments, resource constraints, and evolving healthcare policies while maintaining high standards of patient care. Research underscores that fostering resilience through self‐care, professional support, and reflective practice enhances job satisfaction, staff well‐being, and patient outcomes (Henshall et al. 2020). This ability to endure and adapt ensures that nurses continue to provide high‐quality care despite systemic challenges.

The COVID‐19 pandemic highlighted the critical role of resilience, as nurses worldwide faced unprecedented challenges. The ability to adapt swiftly to new protocols, manage heavy workloads, and sustain emotional strength under immense pressure underscored the enduring dedication to care of those within the profession. In addition to the physical demands of the job, the emotional labour of working in high‐pressure environments has underscored the need for ongoing support, professional development, and leadership (Bond et al. 2022). The risk of compassion fatigue, particularly in critical care settings, became increasingly evident, emphasising the need for resilience‐building strategies (Alharbi et al. 2020).

Drawing from the lessons of the past and challenges of the present is a reminder that resilience in nursing is not just about surviving adversity; it is about using those experiences to strengthen our practice and shape the future of healthcare.

## 3 Beyond Resilience: Deeper Connections to the Work of Caring for Others

Beyond resilience, challenging experiences can also lead to personal and professional growth. The concept of ‘post‐traumatic growth’ refers to the positive psychological changes that can arise after facing significant stress or trauma (Tedeschi and Calhoun 2004). The notion that individuals can experience profound positive transformation when faced with significant life crises and challenges is deeply rooted in history. Scholars like Viktor Frankl (1905–1997), Gerald Caplan (1917–2008), and Irvin Yalom explored this potential for positive transformation during the mid‐20th century (Black and Flynn 2021). This may align with the experiences of many nurses, who, through overcoming difficulties, develop emotional intelligence and a stronger connection to their work.

Studies have shown that post‐traumatic growth in nurses is linked to improved quality of care and better outcomes for both patients and staff (Chen et al. 2021; Itzhaki et al. 2015). When nurses are able to process and grow from difficult experiences, they develop greater empathy, emotional intelligence, and professional insight. With the right support, these qualities can help nurses positively shape team dynamics and enhance patient care (Bond et al. 2022).

Historical pioneers such as Linda Richards championed humane approaches to mental health care, laying the foundation for integrating emotional intelligence in nursing practice. The continued emphasis on both technical expertise as well as relational aspects of care in modern nursing underscores the importance of nurturing emotional well‐being within the profession.

The ability to find meaning in challenges and turn adversity into motivation allows nurses to maintain their commitment to patient care even in the most demanding circumstances. This philosophy continues to guide my practice and scholarship, working to enhance students' clinical competencies as well as the relational aspects of care that are necessary for developing deeper connections (Bond et al. 2024).

## 4 Conclusion

Amidst the challenges of nursing, it is essential to reflect on the motivations that led to choosing the nursing profession. Nurses continuously navigate physical, emotional, and systemic challenges, yet their dedication to patient care remains unwavering. Recognising what fuels resilience and commitment can provide a source of strength during difficult moments.

Intentional efforts, including self‐care, professional support, and ongoing education are required to sustain motivation. Through fostering resilience and embracing opportunities for growth, nurses not only enhance their own well‐being but also contribute to the advancement of compassionate, effective healthcare. As such, staying engaged in lifelong learning, mentorship, and peer support can enable nurses to create a sustainable and fulfilling career path while inspiring future generations to join the profession.

The act of caring is both a professional responsibility and a continuous source of inspiration. As healthcare evolves, staying connected to the core purpose of nursing ensures that the profession remains a source of support and healing for those in need. The ability to balance professional excellence with personal well‐being will continue to shape the future of nursing, ensuring that it remains a deeply rewarding career for years to come.

## Conflicts of Interest

The author declares no conflicts of interest.

## Data Availability

Data sharing is not applicable to this article, as no new data were created or analyzed in this study.
